# Oculogyric crisis in two patients treated with aripiprazole for chronic tics

**DOI:** 10.1007/s10072-026-09056-7

**Published:** 2026-04-23

**Authors:** Andrea E. Cavanna, Chiara Colangelo, Claudia Melina, Annachiara Ravasi, Gabriele Arienti, Anna Riva, Renata Nacinovich, Stefano Seri

**Affiliations:** 1 Department of Neuropsychiatry, BSMHFT and School of Medical Sciences, College of Medicine and Health, University of Birmingham, Birmingham, UK; 2 Sobell Department of Motor Neuroscience and Movement Disorders, Institute of Neurology and University College London, London, UK; 3 School of Health and Life Sciences, Aston Institute of Health and Neurodevelopment, Aston University, Birmingham, UK; 4 School of Medicine and Surgery, University of Milano-Bicocca, Milan, Italy; 5 Department of Child Neuropsychiatry, Fondazione IRCCS San Gerardo dei Tintori, Monza, Italy; 6 Department of Neuropsychiatry, National Centre for Mental Health, 25 Vincent Drive, Birmingham, B15 2FG UK

**Keywords:** Aripiprazole, Oculogyric crisis, Tics, Tourette syndrome

## Abstract

**Background:**

Oculogyric crisis and other acute dystonic reactions have been reported as a rare adverse effect of aripiprazole, a third-generation antidopaminergic medication used for the treatment of multiple neuropsychiatric conditions, including psychotic disorders, affective disorders, obsessive-compulsive disorder, and neurodevelopmental disorders.

**Case description:**

We document two cases (two females aged 16 and 22 years) diagnosed with a neurodevelopmental tic disorder (Tourette syndrome), who developed oculogyric crisis while taking medium/high-dose aripiprazole 20-30 mg daily as a first-line anti-tic agent. Their acute dystonic manifestations completely regressed following dose reduction of aripiprazole.

**Discussion:**

Aripiprazole-induced oculogyric crisis has previously been reported in a total of 18 cases (9 females, age range 11–28 years). Of these, only two (an 18-year-old male and a 21-year-old female) were taking aripiprazole for their tic disorder. In addition to further documenting treatment-emergent oculogyric crisis in patients with Tourette syndrome taking aripiprazole for the treatment of their tics, our reports raise the possibility of dose-dependent mechanisms underlying the development of oculogyric crisis, at least in selected cases.

## Introduction

Aripiprazole is a third-generation antidopaminergic agent whose therapeutic effects are largely attributed to its pharmacodynamic profile, including partial agonist activity at human dopamine D2/D3 and serotonin 5-HT1A receptors, along with antagonistic activity at serotonin 5-HT2A receptors [1]. Its primary clinical indications encompass psychotic disorders, affective disorders, obsessive-compulsive disorder, and tic disorders. Aripiprazole is generally regarded as having a favorable tolerability profile, particularly with respect to extrapyramidal symptoms and metabolic side effects that are frequently observed with other antidopaminergic drugs. Notably, aripiprazole treatment is associated with decreased serum prolactin concentrations and carries a lower risk of QTc interval prolongation [1]. Commonly reported adverse effects include insomnia, akathisia, headache, nausea, vomiting, weight gain, and somnolence. Ocular neuromuscular disorders, including oculogyric crisis, are rare occurrences, mainly documented as isolated case reports [2–16]. We present two patients with a tic disorder (Tourette syndrome, TS) who developed aripiprazole-induced oculogyric crisis. All cases were seen at the specialist Tourette syndrome Clinic, Department of Neuropsychiatry, BSMHFT and University of Birmingham (United Kingdom), for the assessment and management of their motor and vocal tics.

## Case reports

A.B., a 16-year-old girl with a diagnosis of TS, reported the onset of repetitive neck movements at the age of 11. She subsequently developed multiple motor tics affecting her face, trunk and limbs, as well as a repertoire of simple and complex vocal tics including grunting, throat clearing, coughing, barking, humming, shouting, squeaking, blowing raspberries, vowel sounds, and entire words. As part of her complex tic repertoire, she reported coprolalia, copropraxia, echolalia, echopraxia, palilalia, palipraxia, forced touching, and other socially inappropriate behaviors. She was also diagnosed with tic/related obsessive-compulsive disorder (checking, counting, concerns with symmetry and evening-up behaviors). There was a family history of tics (younger brother) and obsessive-compulsive behaviors (both parents). On neurological examination, there was evidence of both motor and vocal tics, namely eye blinking, head jerking, knee movements, and coughing. In addition, she displayed occasional self-injurious tics, including self-hitting, which were characteristically preceded by premonitory urges and suppressible for a few seconds, at the expense of increasing inner tension. She scored 100% on the TS Diagnostic Confidence Index (DCI) and 80% on the Yale Global Tic Severity Scale (YGTSS), indicating marked tic severity. Her pharmacotherapy included aripiprazole 10 mg daily, sertraline 150 mg daily, and promethazine 25 mg daily. There was no history of previous exposure to antidopaminergic agents. In consideration of the severity of her tics, the dose of her aripiprazole was gradually increased in steps of 5 mg every two weeks up to 20 mg daily, however two weeks after reaching her target dose she developed episodes of upward eye rolling lasting for about 15 min, in the absence of premonitory urges. Aripiprazole dose reduction (10 mg daily) resulted in resolution of the oculogyric crisis. No further decreases or switching to different anti-tic pharmacotherapy were required.

C.D., a 22-year-old female, was first noticed as having repetitive head nodding at the age of 7. She subsequently developed multiple motor tics affecting her face, neck, shoulders, trunk and limbs, as well as vocal tics (grunting, ‘ugh’ sounds, sniffing, humming, clicking, and coughing). With regard to complex tics and tic-related symptoms, she reported echolalia, echopraxia, palilalia and palipraxia. She also reported tic-related obsessive-compulsive symptoms, including checking, counting, ‘just right’ perceptions, concerns with symmetry and evening-up behaviors. Both her father and a maternal uncle had motor tics, whereas her older sisters received treatment for anxiety. On neurological examination, there was evidence of both motor and phonic tics, namely blinking, head movements, shoulder shrugging, and sniffing. She scored 73% on the DCI and 51% on the YGTSS, indicating moderate tic severity. She had not previously received pharmacotherapy. In consideration of the impact of her tics on her health-related quality of life, she was prescribed aripiprazole 5 mg daily, which was gradually increased in steps of 5 mg every two weeks up to 30 mg daily, according to response. Three days after reaching her target dose she developed weekly episodes of eye rolling lasting for up to 30 min, which were not preceded by premonitory urges and appeared to be consistent with oculogyric crisis. These adverse effects persisted despite the co-administration of Procyclidine up to 20 mg daily. Symptom remission was achieved following aripiprazole dose reduction (15 mg daily).

## Discussion

To the best of our knowledge, a total of 18 cases of aripiprazole-related oculogyric crisis [2–16] have been reported to date (Table 1).

**Table 1:** Case studies of aripiprazole-induced oculogyric crisis

Case report	Age, sex	Diagnosis	Aripiprazole dose (mg/day)	Treatment duration	Other medications	Intervention	Oculogyric crisis resolution
Fountoulakis et al., 2006 [2]	18 M	Tourette syndrome	10	3 days	Nil	Add-on biperiden	Y
Lim et al., 2008 [3]	23 M	Psychosis	10	9 months	Nil	Dose reduction + add-on procyclidine and lorazepam	Y
Bhachech, 2012 [4]	28 F	Psychosis	20	3 weeks	Eszopiclone	Discontinuation + switch to promethazine	Y
Rizzo et al., 2012 [5]	21 F	Tourette syndrome	7.5	6 days	Fluoxetine	Dose reduction	Y
Gupta and Balhara, 2014 [6]	23 F	OCD	30	< 1 month	Olanzapine	Discontinuation + switch to escitalopram, propranolol, trihexyphenidyl	N
Gardner et al., 2015 [7]	16 F	Psychosis	10	3 months	Risperidone	Discontinuation	Y
Gardner et al., 2015 [7]	22 F	Psychosis	N/A	N/A	Quetiapine, valproate	Add-on niacin	Y
Nebhinani and Suthar, 2017 [8]	19 M	Borderline personality disorder	30	2 weeks	Nil	Dose reduction + add-on trihexyphenidyl	Y
Suthar and Nebhinani, 2018 [9]	19 M	OCD	10	5 days	Fluoxetine	Discontinuation + switch to promethazine	Y
Canol et al., 2020 [10]	14 F	Bipolar disorder	15	2 years	Quetiapine	Discontinuation	Y
Mercan Işık et al., 2020 [11]	11 F	ADHD	2.5	3 days	Methylphenidate	Add-on biperiden	Y
Bernardo et al., 2021 [12]	11 M	ASD	10	1 month	Nil	Dose reduction	Y
Bernardo et al., 2021 [12]	13 F	OCD	15	3 months	Fluoxetine, valproate	Dose reduction	Y
Bernardo et al., 2021 [12]	14 M	ASD	10	2 weeks	Valproate	Add-on trihexyphenidyl	Y
Bafarat et al., 2023 [13]	16 F	Depression	40	1 day	Acetaminophen	Discontinuation + switch to benztropine	Y
Hadler et al., 2023 [14]	19 M	Psychosis	5	3 days	Nil	Discontinuation + switch to diphenhydramine	Y
Malik et al., 2024 [15]	24 M	Depression	10	1 day	Benztropine, duloxetine	Discontinuation + switch to diphenhydramine and quetiapine	Y
Tokioka et al., 2026 [16]	21 M	Depression	24	N/A	Nil	Dose reduction	Y
Present case n. 1 (A.B.)	16 F	Tourette syndrome	20	2 weeks	Promethazine, sertraline	Dose reduction	Y
Present case n. 2 (C.D.)	22 F	Tourette syndrome	30	3 days	Nil	Dose reduction	Y

Abbreviations. *ADHD*, attention-deficit and hyperactivity disorder; *ASD*, autism spectrum disorder; *N/A*, not available; *OCD*, obsessive-compulsive disorder

Half of the patients were females, with ages ranging from 11 to 28 years. Only two out of the 18 patients (an 18-year-old male and a 21-year-old female) were taking aripiprazole for their tic disorder. In the other cases, the main indication was psychosis (*n* = 5), affective disorders (*n* = 4), obsessive-compulsive disorder (*n* = 3), and challenging behaviors in the context of autism spectrum disorder (*n* = 2), attention-deficit and hyperactivity disorder (*n* = 1) and borderline personality disorder (*n* = 1). Most patients developed oculogyric crisis within the first week (*n* = 8) or the first month (*n* = 6) of aripiprazole treatment (dose range 2.5-40 mg daily). Oculogyric crisis was reversible in all reported cases but one - a 23-year-old female who was prescribed high-dose aripiprazole (30 mg daily) for her obsessive-compulsive disorder. In eight patients, their oculogyric crisis resolved following aripiprazole discontinuation. Other strategies leading to symptom resolution included aripiprazole dose reduction (*n* = 6) and co-administration of an anticholinergic agent (*n* = 5).

A real-world study from 2016 to 2022 based on Food and Drug Administration Adverse Event Reporting System database assessed the potential risks of different atypical antipsychotics causing ocular side effects [1]. Although olanzapine had the highest signal intensity in oculogyric crisis, the highest signal strength in overall ocular adverse reactions and blepharospasm was reported for aripiprazole. According to its pharmacodynamic properties, aripiprazole modulates dopaminergic activity but lacks anticholinergic effects, possibly resulting in an increased risk of extrapyramidal side effects, including oculogyric crisis [1, 12]. Moreover, the spectrum of the ocular adverse effects of aripiprazole includes the known correlation between aripiprazole and drug-induced myopia [1, 17].

Our two patients were prescribed aripiprazole with a slow titration schedule. Neither of them had previously been exposed to different antipsychotics or other antidopaminergic agents. Shortly after reaching their target dose (two weeks on 20 mg daily, three days on 30 mg daily), they developed clinical episodes of upward eye rolling lasting for 15–30 min, which differed from their motor tics in key phenomenological aspects. In addition to their episodic nature, the eye rolling spells were not preceded by premonitory urges and were caused by the sustained, spasmodic, tonic contraction of extraocular muscles that characterizes oculogyric crisis.

Beyond providing additional evidence of treatment-emergent oculogyric crisis in patients with TS receiving aripiprazole for tic management, our reports suggest the potential involvement of dose-dependent mechanisms in the pathogenesis of oculogyric crisis, at least in a subset of cases. Further investigation is required to define the precise nature and prevalence of this rare adverse effect associated with aripiprazole, as well as to elucidate its underlying pathophysiological mechanisms across diverse clinical populations.
